# Exploring Needs and Quality of Life of Forensic Psychiatric Inpatients in the Reformed Italian System, Implications for Care and Safety

**DOI:** 10.3389/fpsyt.2020.00258

**Published:** 2020-04-03

**Authors:** Ellen Vorstenbosch, Luca Castelletti

**Affiliations:** ^1^Unitat de Recerca, Docència i Innovació, Parc Sanitari Sant Joan de Déu, Sant Boi de Llobregat, Spain; ^2^Sant Joan de Déu Research Foundation, Barcelona, Spain; ^3^Centro de Investigación Biomédica en Red de Salud Mental (CIBERSAM), Madrid, Spain; ^4^Dipartimento di Salute Mentale, Azienda Ulss 9 Scaligera, Verona, Italy

**Keywords:** forensic psychiatry, needs, quality of life, recovery-oriented treatment, *Residenze per l'Esecuzione delle Misura di Sicurezza*, forensic psychiatric patients, Italy

## Abstract

The Italian forensic psychiatric system underwent drastic reforms. The newly developed facilities are inspired by psychiatric community services, embracing a recovery-oriented approach. Needs and quality of life are broader concepts that consider the more rehabilitative and humanitarian aspects of treatment. In one of the new Italian forensic psychiatric services, this cross-sectional study aimed to investigate the needs and quality of life of forensic psychiatric patients. A second aim was to validate the Italian version of the Forensic inpatient Quality of Life questionnaire Short Version (FQL-SV). Overall, 42 forensic psychiatric patients were assessed using the Forensic version of the Camberwell Assessment of Need (CANFOR), the Historical-Clinical-Risk-Management-20 (HCR-20), the FQL-SV, and the World Health Organization Quality of Life (WHOQoL-Bref). Patients reported significantly fewer needs, whether met or unmet, than their treating clinicians. The general level of agreement between patients and clinicians on specific needs was low Kappa values were < .40 for 64% of the total needs and 46% of the unmet needs. Risk factors according to the HCR-20 mean scores were 13.1, 4.6, and 6.4 for the historical, clinical and risk management subscale. Quality of life was moderate to high for 74% of the patients. Our results showed that lower numbers of needs, whether reported by patients or clinicians, were associated with a better quality of life. The Italian FQL-SV had a Cronbach's alpha of 0.86 and correlated as expected with the WHOQoL-Bref. The FQL-SV is a valid and reliable tool, justifying its use for routinely assessing QoL in Italian forensic psychiatric services. This study enhances our understanding of needs and quality of life of forensic psychiatric patients and how their assessment could have an additional value for recovery-oriented treatment in forensic psychiatry. Although the detained status of forensic patients imposes real limits on the capacity for autonomy and choice, incorporating the patient's perspective on decision-making processes, in relation to aspects of treatment, care, and daily life, may have benefits such as a better treatment adherence or therapeutic alliance. Future research should clarify how routinely assessing needs and quality of life can contribute to the recovery of these forensic psychiatric patients.

## Introduction

In 2008, after a warning from the council of Europe for violation of human rights because of poor quality of care and living conditions, the Italian government approved a major reform of mental health care for forensic psychiatric inpatients. The *Decreto della Presidenza del Consiglio dei Ministri* (DPCM/2008) transferred all responsibilities for general and mental health care both in prisons and the *Ospedali Psichiatrici Giudiziari* (OPG; Forensic Psychiatric Hospitals) from the Ministry of Justice to the National Health Service (NHS). All forensic psychiatric inpatients, hospitalized at that time in the OPGs, were gradually discharged and transferred to ordinary psychiatric NHS settings or newly established *Residenze per l'Esecuzione della Misura di Sicurezza* (REMS) (1). This process got a definite acceleration with Law 81/2014, which established the definite closure of the six national OPGs by the 1^st^ of April 2015 (2). The last patients discharged from an OPG were those in Barcellona P.d.G. (ME) in February 2017. Currently, there are 35 new REMS with security measures that host up to 600 patients (3).

The REMS are intended to better meet the needs of providing intensive and high-quality mental healthcare under proper secure conditions (1). Inspired by psychiatric community services, the REMS are developed as small-scaled (maximum 20 beds) therapeutic environments and built according to the same characteristics and standards as other psychiatric and rehabilitation facilities. Staff are exclusively clinicians and security is provided physically (e.g. fences, locked and secured access, technical devices), relationally (high staff-patient ratio compared to non-forensic units), and procedurally (e.g. risk assessment and management) (4). The common approach in the REMS is recovery-oriented treatment. The emphasis lies on individualized care pathways, including the patients' individual psychosocial and treatment needs, and consideration of the index offense. Treatment is mainly aimed at improvement of insight, understanding of the disorder and its effects, reduction of symptoms, strengthening familiar and services' networks, and ensuring a therapeutic alliance (5).

Recovery-oriented treatment in forensic psychiatry is challenging. It entails engaging patients in their life, on the basis of their own goals and strengths, and supporting them to find meaning and purpose through constructing or reclaiming a valued identity and social roles (6). Patients should be empowered to become self-determined and, hence, be actively involved in decision-making and treatment-planning. Due to the nature of the patient population, their potential risk of recidivism and the restrictiveness of the system and facility, implementing recovery-oriented treatment in forensic psychiatry is complicated (7). Forensic psychiatric patients have mental health difficulties and functional impairment, but also present a history of criminal behavior, violent or sexual offending, a high prevalence of comorbid personality disorder, behavior disturbance, self-harm, and substance use (8). Treatment is thus related to a patient's clinical and psychopathological needs but should also take into account the balance between his/her needs and the needs for safety (9). Given these unique rehabilitative needs, Dorkins & Adshead (10) foresee four problems for the recovery-approach in forensic settings: the values and identity of forensic psychiatric patients, social exclusion as a community response to trauma and violence, empowerment for those who misuse power and do not respect the choices of others, and hopelessness and the offender identity. This limits how much primacy can be given to the perspective of the patient relative to that of professionals (7) and how far recovery-oriented treatment can be fully deployed in forensic psychiatric services.

Notwithstanding, two concepts in line with the recovery-oriented approach that also consider the rehabilitative and humanitarian aspects of treatment are needs and Quality of Life (QoL) (11). In forensic psychiatry, the notion of need has principally been directed by risk reduction and management (12). To reduce the risk of reoffending, treatment focuses on dynamic risk factors directly linked to criminal behaviors (e.g. substance abuse, antisocial personality, pro-criminal attitudes). These dynamic risk factors are referred to as criminogenic needs. In recent years, there has been an emerging interest in a broader understanding of need (13). To ensure comprehensive forensic psychiatric treatment, also general or non-criminogenic needs should be addressed (14, 15). The GLM, for instance, stipulates that non-criminogenic needs such as anxiety, low self-esteem, and psychological distress, should be necessarily targeted to facilitate the learning of new skills or competencies (16). Accordingly, a need can be defined in terms of a difficulty or impairment that requires an interventions to meet it. In other words, a need can be defined as the possibility of benefitting from treatment (17). Studies in forensic psychiatric services have identified treatment needs related to psychotic symptoms and physical health, but also social and relationship-related areas such as daytime activities and company (18–21). QoL, at a minimum, can be defined as an overall “sense of well-being and satisfaction experienced by people under their current living conditions” (22). QoL is a broad concept that encompasses aspects like physical functioning (e.g. ability to perform daily activities), psychological functioning (e.g. emotional and mental well-being), social functioning (e.g. relationships with others and participation in social activities), and perception of health status, pain and overall satisfaction with life (23). Generally, it is considered to consist of objective (resource availability and objective life conditions) and subjective indicators (individual's evaluation of his or her life) (24). Objective and subjective QoL are different constructs. Improvement of objective indicators does not necessarily enhance the subjective evaluation and differences in subjective QoL can not necessarily be explained by objective indicators (25, 26). Studies among forensic psychiatric patients have shown that a better QoL was related to (leisure) activities, living environment and health (27–29).

Overall, the empirical evidence on needs and QoL in forensic psychiatric patients is scarce. Studies in general psychiatry, however, have shown that the concepts seem to be related. Higher levels of unmet needs were associated with lower subjective QoL (30). This association sustained over time and predicted subjective QoL at a one-year follow-up (31). Furthermore, needs and QoL can vary significantly among forensic psychiatric patients, reflecting the wide heterogeneity of this specific psychiatric population (32–34). For instance, lower levels of global functioning were associated with higher numbers of unmet needs (35, 36) and patients with severe mental illness were significantly more satisfied with their QoL than patients with a personality-disorder admitted at the same clinic (37).

The newly developed REMS, with a central role for the patient in his or her treatment and care planning, could profit from systematically assessing needs and QoL. The assessment may help to identify problematic or unsatisfying aspects in a patient's life, to ascertain what aspects can be improved and eventually monitor the patient's progress. The patient's perception of their daily lives, their experiences in the REMS and their perception of these experiences (38), are key in their willingness to change. Patients shall be reluctant to change aspects in life they are satisfied with, whereas not addressing aspects they are unsatisfied with might jeopardize the therapeutic alliance (39). Disagreement on needs and QoL outcomes can be an indication for the need to negotiate treatment goals. Treatment focused on needs and QoL favors the individual approach and monitoring their outcomes fosters tailoring interventions within particular domains. Additionally, discussing needs and QoL on a regular basis supports the dialogues between patient and clinician, betters the therapeutic alliance and even enhances the patient's experienced QoL (40, 41). Systematic assessment of treatment needs and QoL may provide information for treatment planning in addition to other relevant outcomes such as the risk of criminal recidivism, reduction of psychiatric symptoms, psychological functioning, etc.

The Italian forensic reform stresses the importance of developing pathways of care at low levels of therapeutic security and focused on recovery-based determinants. Rooted in Articles 3 and 46 of the National Constitution, it affirms the primacy of health, physical and mental rights for citizens, as well as the duty of the Republic to guarantee proper treatments in adequate environments for all its citizens. In this light, assessment of needs and QoL assumes a priority task to measure the quality of services, and the capacity to target therapeutic programs. Moreover, it provides complementary information for management decisions, the type of treatment and/or the most suitable facility (42). Finally, the Italian forensic psychiatric system and its recent reforms have been described extensively (2, 43). However, the lack of studies supported by data is considered an eminent gap within the Italian system (5), and several authors stress the importance of systematically collecting data for service evaluation (1, 44).

This study, therefore, aimed to present the first results of a comprehensive set of measurements to routinely monitor recovery-oriented treatment at the Veneto REMS in Nogara (VR). More specifically, 1) we present the results of needs and QoL assessment, and 2) their relationship with other concepts such as risk assessment and global functioning. A third aim was to assess the validity of the Italian translation of the FQL-SV, an instrument developed for the assessment of QoL within forensic psychiatric inpatient services.

## Materials and Methods

### Study Setting and Data Collection

The study was conducted at the Veneto REMS in Nogara (VR), Italy. This REMS has been open for admission since January 2016 and is functioning in its full capacity since June 2016. The REMS offers forensic psychiatric care to male and female adults from the Veneto region, covering approximately a catchment area of 4.9 million people. The Veneto REMS is hosted in a Verona NHS building, a former small suburban hospital, now mainly converted into an outpatient service. The wards of the REMS are taken from previous inpatient general medical service, with a fenced garden open during daytime for most of the patients. The Veneto REMS has a capacity of 40 beds, divided over two wards. Rooms are mainly single and double and are unlocked all day round. The current facility is temporary, as the Veneto Region is planning to entirely renovate the in- and outdoor spaces from near facilities.

The population is essentially composed of patients from the Veneto region, except for homeless people, in that case, the crime site defines the place of admission. Those admitted are generally convicted for a serious offense, and deemed by local courts not responsible for the index delict for reason of insanity (Art. 88 c.p.) or alternately considered partially responsible (Art. 89 c.p.), for which a psychiatric security measure is applied at the end of the correctional penalty, generally reducing its length one third. All admitted patients have an Axis one diagnosis and frequently comorbidity on Axis II, defined according to the DSM-5 (45). The majority of the patients are well-known by psychiatric community services, and only a few have never been in contact with the local mental health services. Those who were previously known often had a difficult engagement with services and many of them had at least one community treatment order (*Trattamento Sanitario Obbligatorio*) to recover from a severe mental state and personal unavailability to be treated.

This study is part of ongoing routine outcome measuring (ROM) in a cohort of Italian forensic psychiatric inpatients residing at the Veneto REMS and should be considered as a first measurement. The data for the current study were collected between June 2018 and July 2019. The assessments were conducted within the framework of routine care and treatment planning by the patients' treating key-clinicians. To let patients become aware of his/her current QoL and needs profile, and the staff to collect enough information, assessments took place between the 3rd and 12th month after admission. At the time of the study, seven patients (17%) had not completed the CANFOR interview; two were discharged before the interview could take place and five were only recently admitted. Completion of the QoL instruments was supported by the clinician in case a patient suffered dyslexia, illiteracy, or poor concentration; otherwise, patients were asked to complete the questionnaires by themselves. Clinical and demographic data were obtained from file reviews by one of the key-treating clinicians (L.C.). Socio-demographic and clinical variables such as primary diagnoses were collected from the REMS' register of admissions. Scores on the Global Assessment of Functioning (GAF) (45) and the HCR-20 Violence Risk Assessment Scheme (HCR-20^V3^) (46) are part of the ROM dataset established at the REMS. Information about index offenses was derived from the criminal register.

For the purpose of the reliability and validity analyses of the Italian version of the FQL-SV, the data from the Veneto REMS were combined with data from the OPG in Castiglione delle Stiviere (Mantua, Lombardy). The data from the OPG were collected in December 2015 when the OPG had just started its reforms. At that time, only one unit met the requirements of a REMS. However, due to its small scale, this unit was being used as an admission ward; therefore, the data from this ward were excluded from analyses. At the remaining wards resided 40, 70, and 50 patients, respectively. Patients were invited to participate by their treating clinicians, either the patient's psychologist or psychiatrist. The exclusion criteria were: inability to complete the questionnaires due to psychotic episodes and/or major chance of decompensation as judged by the clinician, insufficient mastery of the Italian language or seclusion. Overall, 70 patients were approached; 54 (76.2%) were willing to participate and 16 (22.8%) refused. In case a patient suffered dyslexia, illiteracy or poor concentration, completion of instruments was supported by the clinician, otherwise, the patient was asked to complete the questionnaires by themselves.

#### Ethical Considerations

Since assessments were conducted within the framework of routine care by patients' treating key-clinicians, approval was sought from the Clinical Directors. Privacy of the patients and clinicians was assured conform the policy of the institutions. Data were transferred to the researchers in a fully anonymized form; therefore, all statistical analyses were conducted on fully anonymous data. Written informed consent was provided by all participants and all patients were informed of their right to withdraw consent at any time. The study was approved by the *Comitato Etico di Verona* (Ethics Committee of Verona).

### Variables and Instruments

#### Needs

Needs were assessed with the Forensic version of the Camberwell Assessment of Need (CANFOR) (47). The CANFOR is designed to identify the needs of forensic psychiatric patients. It is considered to be a valid and reliable needs assessment instrument (20, 48) and has been translated and validated for use in forensic psychiatric services in Spain, Portugal and Italy (19, 36, 49). Through a partially structured interview, the CANFOR integrates the patient's and clinician's perspective on 25 domains of frequent or important problem areas for forensic patients. If there have been no difficulties in a particular area, a need is scored as not present (score “0”). If there were some difficulties in a certain area, the need can either be met or unmet. A met need means that due to an appropriate intervention there are currently no difficulties in that area (score “1”). An unmet need means that no interventions are currently being provided or that the provided interventions are not perceived as effective; there are currently serious difficulties in that area (score “2”). The total need score is the sum of the number of identified met and unmet needs (scores “1” and “2”). If a need is not considered to be present, it can be scored as no need (score “0”) or, in certain instances, not applicable (code “8”) or not known (code “9”). For the purpose of analysis, these scores were combined because some clinicians rated no problem (score “0”) when they did not know about a patient's need, whereas others rated not applicable or not known (code “8” or “9”). Any differences between the patient and clinician in the perception of a need are apparent by directly comparing the scores.

#### Violence Risk Factors

Violence risk factors (i.e. criminogenic needs) were assessed with the Italian version of the Historical-Clinical-Risk-Management-20 Version 3 (HCR-20^V3^) (50). The HCR-20 is a structured instrument that assesses the potential risk of violence and has demonstrated to be a reliable and valid instrument in forensic psychiatric populations (51, 52). The HCR-20^V3^ contains 20 items that are divided over three subscales: 10 historical items, five clinical items, and five risk management items. The historical items are fixed and non-modifiable, conversely clinical and risk management are dynamic, can change with the evolution of the patient's state. Each item is judged by a professional and rated according to whether it is present (score “2”), possibly or partially present (score “1”) or absent (score “0”). The total HCR-20^V3^ and three subscales are calculated based on the sum of these scores, The HCR-20 has shown to be a valid risk assessment instrument in forensic psychiatric populations (53) and findings support the concurrent validity and interrater reliability of Version 3 of the HCR-20 (54).

#### Quality of Life (QoL)

QoL was measured using a translation of the Forensic inpatient Quality of Life-Short Version (FQL-SV) (28) and the Italian version of the World Health Organization Quality of Life Brief Version (WHOQOL-Bref) (55). The FQL-SV is an abbreviated form of the FQL (29), developed for the assessment of QoL within a forensic psychiatric inpatient setting. The FQL-SV consists of 18 QoL items plus one item on acceptance of living in a secure unit for some time, which are all scored on a 100-millimeter VAS-scale. Patients are asked to indicate their level of agreement with the specific item (0=total disagreement; 100=total agreement). The total FQL-SV is based on the mean score of the 18 QoL items. The FQL-SV has shown good psychometric properties (e.g. Cronbach's alpha of.79) (56). Together with a contributor (S.G.), one of the authors (L.C.) translated the English version of the FQL-SV into Italian. A back translation was performed by another contributor (G. T.) and checked for consistency by the first author, who also developed the original FQL (E.V.). Based on a revision with 3 clinicians and the authors, some minor changes were made to meet the reality of the OPG setting in Castiglione delle Stiviere. Specifically, one sub-item was added in the sociodemographic part regarding the number of patients in the department; item 10 about the received opportunities concerning sexuality was simplified into “Are you satisfied with your sexual life?” because the initial wording was considered ambivalent.

The WHOQoL-Bref (57) consists of 26 items measured with a five-point Likert scale. The WHOQOL-Bref is considered reliable among male adults in a forensic psychiatric hospital with Cronbach's alphas ranging between.77 and.79 for the domains and.80 for the total WHOQOL-Bref (58). Following the criteria of the World Health Organization, four domains, namely Physical health, Psychological health, Social relations, and Environment, were calculated and transformed to a 0–100 scale (59). Due to the restricted environment of the REMS, the participating patients cannot make use of public transportation; therefore, item 25 of the WHOQoL-Bref was excluded from this study.

### Analyses

Basic descriptive analyses were conducted to describe the sample's socio-demographic, clinical and forensic characteristics, treatment needs, risk factors, and QoL. To evaluate differences in these variables independent-sample t-tests and analyses of variance (ANOVA) or Mann-Whitney U tests and Kruskal-Wallis tests were used, depending on whether the distribution of variables was normal or non-normal as determined by the Shapiro–Wilk test.

Intra-class correlation coefficients (ICC) were calculated, using a two-way mixed model defining, to assess inter-rater reliability for the CANFOR total needs, total met needs and total unmet needs, as recommended by Leese (2001) (60). Cohen's Kappa coefficients were calculated to assess the level of agreement on each need domain between patients and clinicians. Each CANFOR item was recoded into two domains: identified need (whether met or unmet) and identified unmet need. According to Landis and Koch (1977) (61), Kappa coefficient results to be poor (< 0.21), fair (0.21–0.40), moderate (0.41–0.60), good (0.61–0.80) and very good (0.81–1.0).

Since the Italian version of the FQL-SV had not been validated yet, we explored its internal consistency and construct validity. Internal consistency was examined by calculating Cronbach's alpha (62); a Cronbach's alpha greater than 0.7 has been considered satisfactory (63). Construct validity was assessed by calculating Pearson correlations between the items of the FQL-SV and the domains of the WHOQoL-Bref. Both instruments are intended to measure the same underlying construct, therefore, we expected to find moderate to strong correlations between the FQL-SV items and the WHOQoL-Bref domains. However, the FQL-SV is developed specifically for use in a forensic psychiatric inpatient setting. Hence, we expected to see discrepancies as well. Pearson's correlations of.10–.30 were seen as weak,.30–.50 moderate and > .50 strong (64).

Due to deviations of normality of some variables, Spearman correlation coefficients were used to assess the relationship between needs (CANFOR), QoL (FQL-SV), risk (HCR-20^V3^) and clinical variables. Results were considered significant using the default of *p*=0.05 or lower. The data analyses for this paper were generated using SAS software, Version 9.4 and SPSS Statistic, Version 23.

## Results

### Participants

In this study, 42 forensic psychiatric inpatients consented to participate and 35 of them completed all assessments. The majority of the participants were male (88.1%; n=37) and the mean age was 42 years (range 22–62). At the moment of assessment, patients resided on average 44 months in forensic psychiatric services (range 2–360). Female patients were significantly older than male patients (mean ± SD=50.0 ± 5.8 vs. 40.6 ± 11.0 years, *p*=0.02), but did not differ significantly regarding admission time.

All patients had one diagnose on Axis I and frequent comorbidity with an Axis II diagnosis (n=26, 61.9%). With respect to their primary diagnosis, the most frequent diagnosis was schizophrenia (n=28; 66.7%), followed by personality disorder (n=8; 19.0%) and organic psychoses (n=6; 14.3%).

GAF scores ranged from 26 to 58 with a mean (± SD) score of 44.1 (± 7.8). No significant differences were found in age, time of admission or GAF-score for primary diagnoses. The majority of patients had a substance abuse diagnosis (n=26; 61.9%). Patients with a diagnosis of substance abuse were significantly younger than those who were not diagnosed as such (38.4 ± 9.9 vs. 46.3 ± 10.5, *p*=0.03); no significant differences were found with respect to admission time or GAF score.

Concerning index offenses, the vast majority had committed an offense against a person (n=32; 76.2%). More specifically, 17 patients committed physical abuse (40.5%), nine homicide (21.4%), and six were convicted for attempted murder (14.3%). The rest of the patients (n=10; 23.8%) committed other offenses such as arson, stalking, burglary or robbery. No significant differences were found for index offense with respect to age, time in forensic psychiatric services and GAF-score. At the time of this study, not all instruments were registered for all patients; hence, the number of patients who completed the CANFOR, FQL-SV, WHOQoL-Bref, and HCR-20^V3^, is included in **Table 1**.

**Table 1:** Characteristics of patients admitted to the Veneto REMS (N=42).

	n (%)	Min-Max
Women	5 (11.9)	
Age *(Mean±SD)*	41.7±10.9	22-62
*Country of birth*		
Italy	29 (69.0)	
Germany	2 (4.8)	
Morocco	2 (4.8)	
Romania	2 (4.8)	
Other	7 (16.7)	
*Education*		
Primary school	4 (9.5)	
Secondary school	22 (52.4)	
High school	14 (33.3)	
Degree—University degree	2 (4.8)	
*Primary diagnosis*		
Schizophrenia	28 (66.7)	
Personality disorder	8 (19.0)	
Organic psychoses	6 (14.3)	
Comorbid diagnosis on Axis II	23 (54.8)	
Diagnosis of substance abuse	26 (61.9)	
GAF score *(Mean±SD)*	44.1±7.8	26-58
*Index offense*		
Physical abuse	17 (40.5)	
Homicide	9 (21.4)	
Attempted murder	6 (14.3)	
Other (e.g. arson, stalking, burglary, robbery)	10 (23.8)	
Number of other patients at the unit	19	
Number of patients to share bedroom with	1	0-2
Months in forensic psychiatric services *(Mean±SD)*	43.9 ± 65.8	2-360
Previous contact with local mental health services	34 (80.9)	
At least one community treatment order	26 (61.9)	
*Instruments*		
CANFOR	35 (83.3)	
FQL-SV	42 (100)	
WHOQoL-Bref	37 (88.1)	
HCR-20^V3^	42 (100)	

SD, standard deviation.

### Needs

#### Needs

**Figure 1:**
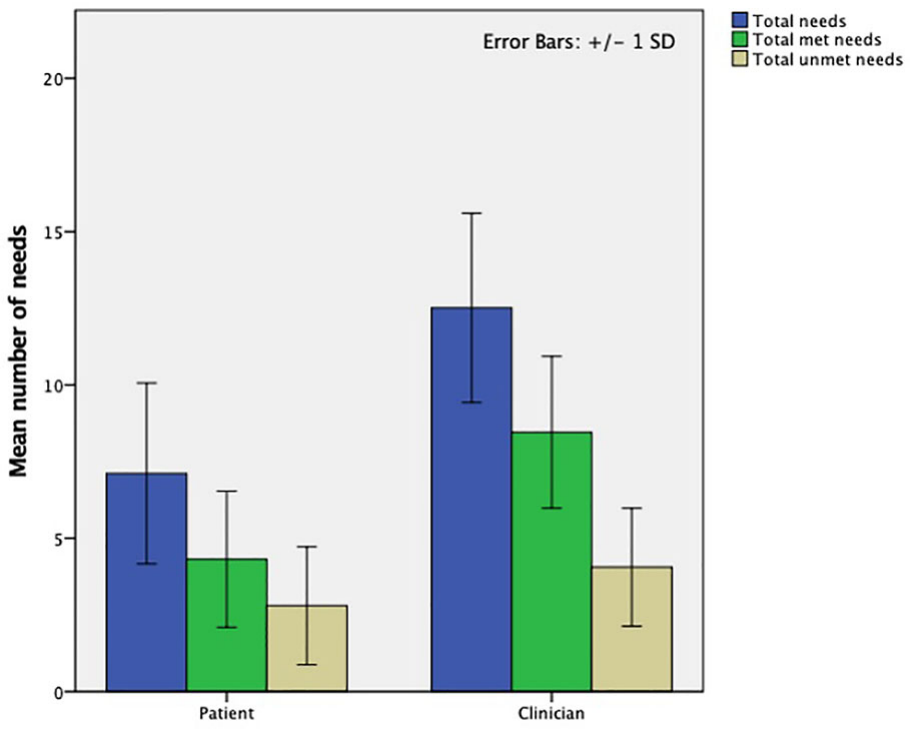
Results of the CANFOR needs assessment: mean number of total, met and unmet treatment needs identified by patients and clinicians at the Veneto REMS (N=35). CANFOR, Camberwell Assessment of Need; REMS, *Residenze per l'Esecuzione della Misura di Sicurezza*.

The outcomes of the CANFOR assessment are presented in **Figure 1**. It shows the mean (± SD) number of total, met and unmet needs reported by patients and their clinicians. Compared to their clinicians, patients reported a significant lower number of total needs (12.5±3.1 vs. 7.1±2.9; *p* < 0.01), met needs (8.5±2.5 vs. 4.3±2.2; *p* < 0.01) and unmet needs (4.1±1.9 vs. 2.8±1.9; *p* < 0.01).

**Table 2** shows for each CANFOR domain the number and percentages of needs (regardless of whether the need was met or unmet) and unmet needs identified by patients and their clinicians as well as the corresponding level of agreement and Kappa coefficient. The most common need reported by patients was daytime activities (71.4%; n=25), followed by psychological distress (60.0%; n=21) and benefits (57.1%; n=20). The most common needs according to clinicians were psychological distress (97.1%; n=34), accommodation (94.3%; n=33), and daytime activities, psychological symptoms and company (all 91.4%; n=32). Intimate relationships (42.9%; n=15), benefits (40.0%; n=14) and company (39.4%; n=13) were the needs that were most frequently reported as unmet by patients. According to clinicians, these were accommodation (74.3%; n=26), intimate relationships (54.3%; n=19) and company (51.4%; n=18).

**Table 2:** Results of the CANFOR needs assessment at the REMS in Veneto (N=35).

	Total need^a^				Unmet need			
	Patients^b^ n (%)	Clinicians n (%)	Agreement %	Kappa (SE)	Patients^b^ n (%)	Clinicians n (%)	Agreement %	Kappa (SE)
1. Accommodation	19 (53.3)	33 (94.3)	61.8	0.15 (0.10)	8 (22.9)	26 (74.3)	38.2	0.01 (0.10)
2. Food	7 (20.0)	16 (45.7)	68.6	0.34 (0.14)	1 (2.9)	5 (14.3)	88.6	0.30 (0.24)
3. Living environment	12 (34.3)	21 (60.0)	74.3	0.52 (0.12)	1 (2.9)	–	97.1	NA
4. Self-care	1 (2.9)	15 (42.9)	60.0	0.08 (0.07)	–	2 (5.7)	94.3	NA
5. Daytime activities	25 (71.4)	32 (91.4)	80.0	0.38 (0.16)	6 (17.1)	15 (42.9)	68.6	0.31 (0.14)
6. Physical health	15 (42.9)	13 (37.1)	77.1	0.53 (0.15)	2 (5.7)	–	94.3	NA
7. Psychotic symptoms	15 (42.9)	32 (91.4)	52.9	0.14 (0.08)	3 (8.6)	1 (2.9)	94.1	0.48 (0.31)
8. Information	13 (37.1)	31 (88.6)	48.6	0.14 (0.07)	6 (17.1)	6 (17.1)	71.4	-0.01 (0.17)
9. Psychological distress	21 (60.0)	34 (97.1)	57.1	-0.06 (0.05)	7 (20.0)	11 (31.4)	71.4	0.26 (0.17)
10. Safety to self (self-harm)	4 (11.4)	8 (22.9)	82.9	0.41 (0.19)	2 (5.7)	1 (2.9)	97.2	0.65 (0.32)
11. Safety to others (violence)	5 (14.3)	10 (28.6)	74.3	0.26 (0.17)	2 (5.7)	–	94.3	NA
12. Alcohol	7 (20.0)	19 (54.3)	60.0	0.24 (0.12)	1 (2.9)	2 (5.7)	97.2	0.65 (0.32)
13. Drugs	9 (25.7)	16 (45.7)	80.0	0.58 (0.13)	1 (2.9)	–	97.1	NA
14. Company	18 (51.4)	32 (91.4)	54.6	0.01 (0.09)	13 (37.1)	18 (51.4)	60.6	0.23 (0.16)
15. Intimate relationships	16 (45.7)	21 (60.0)	74.9	0.49 (0.14)	15 (42.9)	19 (54.3)	77.1	0.55 (0.14)
16. Sexual oppression	13 (37.1)	17 (48.6)	90.9	0.82 (0.10)	11 (31.4)	17 (48.6)	84.9	0.69 (0.12)
17. Childcare	5 (14.3)	6 (17.1)	91.4	0.68 (0.17)	4 (11.4)	6 (17.1)	88.6	0.54 (0.20)
18. Basic education	5 (14.3)	7 (20.0)	94.3	0.80 (0.13)	1 (2.9)	1 (2.9)	100	1.00 (0.00)
19. Telephone	4 (11.4)	–	88.2	NA	–	–	100	NA
20. Transport	–	14 (40.0)	60.0	NA	–	–	100	NA
21. Money	5 (14.3)	18 (51.4)	62.9	0.27 (0.11)	–	2 (5.7)	94.3	NA
22. Benefits	20 (57.1)	22 (62.9)	82.9	0.64 (0.13)	14 (40.0)	9 (25.7)	62.9	0.18 (0.16)
23. Treatment	9 (25.7)	22 (62.9)	62.9	0.34 (0.11)	–	1 (2.9)	97.1	NA
24. Sexual offences	–	–	100	NA	–	–	100	NA
25. Arson	–	1 (2.9)	97.1	NA	–	–	100	NA

^a^‘Total needs' includes met and unmet needs (CANFOR score “1” and “2”). “Need not present, not applicable and not known (CANFOR score “0” and code “8” and “9”, respectively) were considered as no need; ^b^For the patient-rated needs there were some missing cases (i.e. Accommodation, Psychotic symptoms, Telephone, Sexual offences (one missing case), Company and Sexual oppression (two missing cases); SE: Standard Error; NA: Not applicable test because the domain was rated as no need category by either the patients or clinicians, or both.

Kappa coefficients for the CANFOR domains showed that agreement on the total needs was very good for two domains (8%; sexual oppression, basic education), good for two domains (8%; childcare, benefits), moderate for 5 domains (20%; living environment, physical health, safety to self, drugs, intimate relationships), fair for six domains (24%; food, daytime activities, safety to others, alcohol, money, treatment) and poor to none for six domains (24%; accommodation, self-care, psychotic symptoms, information, psychological distress, and company). The agreement for unmet needs was very good for one domain (4%; basic education), good for three domains (12%; safety to self, alcohol, sexual oppression), moderate for three domains (12%; psychotic symptoms, intimate relationships, childcare), fair for four domains (16%; food, daytime activities, psychological distress, company) and poor to none for two domains (8%; accommodation, benefits).

#### Violence Risk Factors

**Figure 2:**
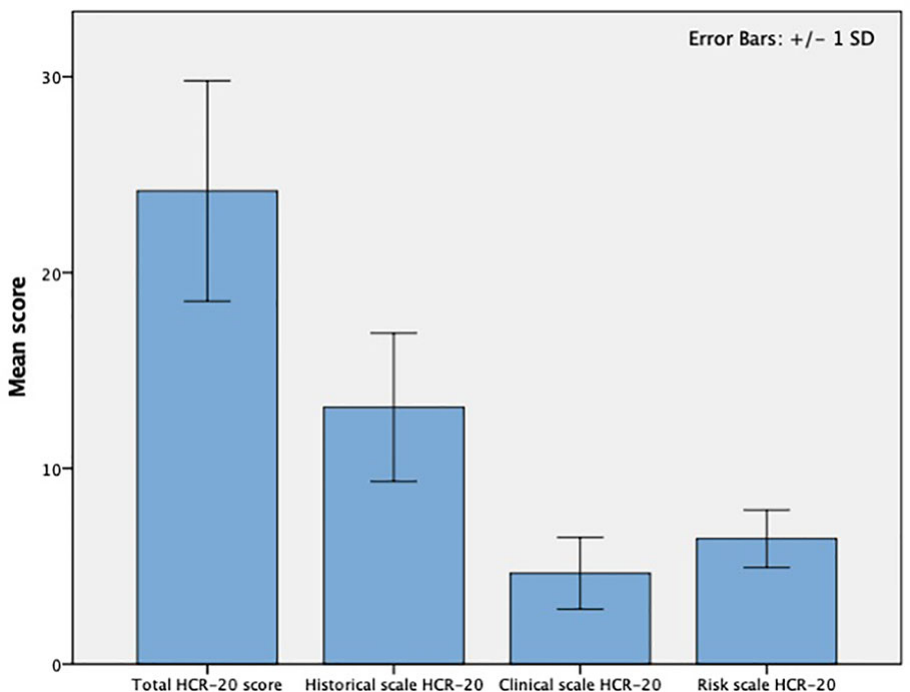
Results of the HCR-20 risk assessment at the Veneto REMS (N=42). HCR-20, Historical-Clinical-Risk-Management-20; REMS, *Residenze per l'Esecuzione della Misura di Sicurezza*.

The mean (±SD) scores on the HCR-20^V3^ and its subscales are presented in **Figure 2**. The mean (± SD) total HCR-20 score was 24.2±5.6 (range 13-36). The mean (± SD) scores on the HCR-20 subscales were as follows: historical items 13.1±3.8, clinical items 4.6±1.8, and risk items 6.4±1.5.

### Quality of Life

#### Internal Consistency and Construct Validity of the Italian FQL-SV

The internal consistency and construct validity of the Italian FQL-SV were tested with data from 91 patients from two forensic psychiatric clinics: the Veneto REMS (n=37) and the Castiglione delle Stiviere OPG (n=54). No significant differences were found between the REMS and OPG populations with respect to gender (women 13.5% vs. 27.8%), age (mean ± SD=41.8 ± 11.4 vs. 39.1 ± 11.9 years) and admission time (mean ± SD=48.9 ± 68.6 vs. 28.1 ± 31.5 months; all *p* > 0.05).

The Cronbach's alphas of the FQL-SV and WHOQoL-Bref were 0.86 and 0.79, respectively. In general, the FQL-SV items and WHOQoL-Bref domains are associated in a coherent and expected manner (**Table 3**). For example, social relations, other residents, daily staff, and affection as well as sexuality correlated positively and significantly with the WHOQoL-Bref social relations domain, meaning that a more positive evaluation on the social items of the FQL-SV was related to a higher appraisal of the social relations as assessed with the WHOQOL-Bref. Residence 1—feeling safe, and 2—pleasant environment, daily staff and affection correlated strongly with the WHOQoL-Bref domain environment. This means that patients who felt safe at the unit, considered to live in a pleasant environment and those who were positive about their daily contact with staff were also more satisfied with their environment according to the WHOQoL-Bref assessment. Finally, the FQL items nutrition, hygiene, and self-actualization showed weak correlations, as these are not considered in the WHOQoL-Bref.

**Table 3:** Construct validity; correlations between subscales of the FQL-SV and WHOQOL-Bref (N=91).

	*WHOQoL-Bref domains*			
	Physical	Psychological	Social relations	Environment^a^
*FQL-SV items*				
1. Activities	.31**	.34**	.19	.27**
2. Leave^b^	.28*	.16	.25*	.32**
3. Residence 1 (Safety)	.17	.13	.17	.51**
4. Residence 2 (Pleasant environment)	.26*	.20	.16	.51**
5. Nutrition	.01	.13	.02	.13
6. Hygiene	.20	.04	-.02	.19
7. Health 1 (Mental health treatment)	.39**	.25*	.38**	.44**
8. Health 2 (Overall health)	.32**	.32**	.18	.17
9. Sexuality	.16	.26*	.63**	.18
10. Social relations	.24*	.32**	.34**	.37**
11. Other residents	.33**	.19	.34**	.40**
12. Daily staff	.40**	.28**	.34**	.55**
13. Affection	.31**	.31**	.44**	.53**
14. Autonomy 1 (Move freely)	.34**	.33**	.18	.49**
15. Autonomy 2 (Make own decisions)	.24*	.25*	.20	.33**
16. Self-actualization	.18	.23*	.21	.10
17. Religion	.32**	.30**	.45**	.30**
18. Overall QoL	.40**	.55**	.53**	.40**

Pearson correlations: ** Correlation is significant at the 0.01 level (two-tailed), * Correlation is significant at the 0.05 level (two-tailed).

Moderate and strong correlations are shown in bold typeface (r ≥.3).

a Item 25 of the WHOQoL-bref has been excluded as it assesses access to public transport, which is not applicable for this population.

b Twenty-two patients skipped the FQL-SV item regarding satisfaction with their current leave status (n=79).

#### FQL-SV QoL Assessment

**Figure 3:**
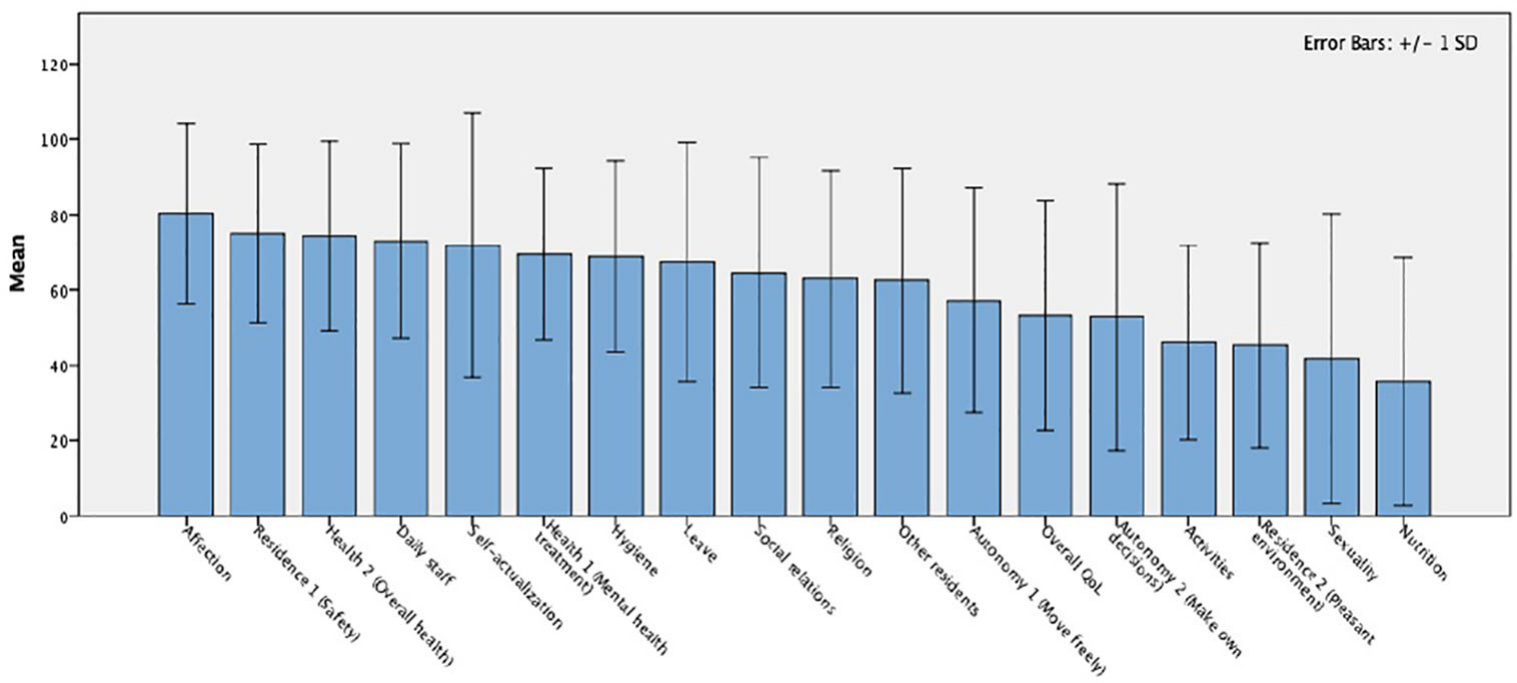
Mean scores of the FQL-SV QoL assessment at the Veneto REMS (N=42); Seven patients skipped the item regarding satisfaction with current leave status (n=34). FQL-SV, Forensic inpatient Quality of Life questionnaire Short Version; QoL, Quality of Life; REMS, *Residenze per l'Esecuzione della Misura di Sicurezza*.

The outcomes of the QoL assessment are presented in **Figure 3**; it shows the mean (± SD) scores on the QoL items assessed with the FQL-SV. The mean (± SD) score on the total FQL-SV was 63.9 ± 16.7 (ranging from 34.2 to 94.4). The aspects patients were most satisfied with were affection (83.5 ± 3.4), daily staff (76.4 ± 3.7) and health 2—overall health (75.3 ± 4.1). Overall, four aspects had a mean score below 50, signifying that patients were unsatisfied with sexuality (39.8 ± 6.3), nutrition (42.1 ± 5.3), residence 2—pleasant environment (48.3 ± 4.7) and activities (49.8 ± 4.5).

Considering equal percentiles for low, moderate and high QoL, 26% of the patients at the Veneto REMS reported a low QoL (FQL-SV < 51.3), 31% a moderate QoL (51.3≤FQL-SV < 65.9), and 43% a high QoL (FQL-SV≥65.9). The FQL-SV also includes one item on acceptance of living in a secure unit for some time. This is not part of QoL measurement but considered important in the context of forensic psychiatric treatment. The mean (± SD) score on this item was 70.4(± 34.8).

### Relationship Treatment Needs, Risk Factors and Quality of Life

Spearman rank-order correlations were calculated to explore the relationship between needs (CANFOR), risk factors (HCR-20^V3^), QoL (FQL-SV) and clinical variables such as acceptance of residing in a forensic psychiatric unit for some time, length of admission, and global functioning (GAF). The correlation matrix showing the significant relationships is presented in **Table 4**. Summarized, the significant results showed that patient-reported unmet needs correlated positively with the HCR-20^V3^ historical items. With respect to clinician-reported needs, the total and unmet needs (CANFOR) correlated positively with the total HCR-20^V3^ and the historical and clinical subscales whereas met needs (CANFOR) correlated only positively with the historical and clinical HCR-20^V3^ subscales.

**Table 4:** Correlation matrix of the relationships between the CANFOR-S, FQL-SV, HCR-20 and clinical variables (N=42).

		1	2	3	4	5	6	7	8	9	10	11	12	13	14
1	CANFOR Total needs (Patient)	–													
2	CANFOR Met needs (Patient)	.73**	–												
3	CANFOR Unmet needs (Patient)	.67**	.03	–											
4	CANFOR Total needs (Clinician)	.47**	.16	.46**	–										
5	CANFOR Met needs (Clinician)	.39*	.21	.28	.76**	–									
6	CANFOR Unmet needs (Clinician)	.23	-.04	.38*	.57**	-.04	–								
7	Total HCR-20^V3^	.25	.04	.29	.53**	.26	.46**	–							
8	HCR-20^V3^ Historical scale	.30	.06	.36*	.61**	.39*	.41*	.93**	–						
9	HCR-20^V3^ Clinical scale	.16	-.01	.20	.53**	.34*	.37*	.82**	.70**	–					
10	HCR-20^V3^ Risk scale	-.00	.03	-.09	-.10	-.26	.18	.39*	.12	.21	–				
11	FQL-SV	-.40*	-.07	-.47**	-.64**	-.29	-.58**	-.59**	-.54**	-.49**	-.27	–			
12	Acceptance of stay	-.09	.10	-.17	-.39*	-.14	-.37*	-.13	-.13	-.20	.03	.59**	–		
13	Length of admission	.29	-.02	-.02	.10	.03	.16	.17	.15	.21	.00	-.14	-.15	–	
14	GAF	-.15	-.16	-.01	-.17	-.15	-.13	-.39**	-.24	-.63**	-.13	.01	-.22	-.23	–

Spearman rank order correlations: ** Correlation is significant at the 0.01 level (two-tailed), * Correlation is significant at the 0.05 level (two-tailed).

Moderate and strong correlations are shown in bold typeface (r ≥.3).

Patient and clinician scores on the CANFOR (n = 35); FQL-SV, HCR-20^V3^ (sub-) scale(s) and clinical variables Acceptance of stay, Length of admission and GAF (n=42).

Total and unmet needs (CANFOR), reported by either the patient or the clinician correlated negatively with QoL (FQL-SV). No such relation was seen for met needs. Concerning risk factors, as assessed with HCR-20^V3^, the total score and the clinical and historical subscale correlated negatively with QoL (FQL-SV). This was not found for the HCR-20^V3^ risk subscale, which did correlate negatively but that correlation was non-significant.

Clinician-reported total and unmet needs (CANFOR), furthermore, correlated negatively with acceptance of residing in a forensic psychiatric unit for some time. QoL (FQL-SV), on the other hand, correlated positively with acceptance of stay. Needs, risk factors, nor QoL correlated significantly with the length of admission in forensic psychiatric services. Finally, only risk factors in the form of the total HCR-20^V3^ score and the clinical subscale correlated negatively with global functioning (GAF).

## Discussion

### Main Findings

This study presents the first outcomes of needs and QoL assessment among forensic psychiatric patients admitted to one of the newly-developed *Residenze per l'Esecuzione della Misura di Sicurezza* (REMS) in Italy. To our knowledge, this is the first study in the reformed Italian forensic facilities investigating these concepts in a structured way. The present study employed comprehensive measures for the evaluation of recovery-oriented treatment such as needs (CANFOR) and quality of life (FQL-SV) and investigated the interrelationship between the CANFOR, FQL-SV, and measures of risk factors (HCR-20^V3^) and clinical variables such as global functioning.

Concerning total needs, the patients at the Veneto REMS reported a comparable number as their counterparts in a medium-security hospital in the United Kingdom and secure mental health services in Australia (15, 20). In our study, however, the proportion of unmet needs was slightly lower (7.1 needs, of which 2.8 were unmet). It means that 39% of the areas in which patients experience difficulties have not yet been resolved 3 to 12 months after admission to the REMS. The areas of unmet needs were similar to those in other studies (15, 18); namely, those in the personal and social areas such as intimate relationships and company. Contrary to previous studies (15, 18–20), our patients reported benefits as a common and unmet need, meaning that they experience difficulties with the financial support they are entitled to and that the help they currently receive is insufficient. The clinicians in our study reported a mean of 12.5 needs, of which 4.1 were considered as unmet. Although these numbers are considerably higher than in earlier European studies (15, 18–20), a recent Australian study by Adams and colleagues (65) showed that the total needs were comparable to patients residing in open or low-security facilities (13.2 and 13.5, respectively). However, the number of unmet needs was closer to that of patients residing in high security (4.6). Psychological distress, accommodation, daytime activities, psychotic symptoms, and company were the most common needs whereas accommodation, intimate relationships and company were most often considered as unmet. These results were largely in line with other studies (15, 19, 20, 49, 66). The practice of violence risk assessment and management has recently been introduced in Italy, concurrently with the reformed system (3). The risk factors in our study, measured with the HCR-20, were characteristic for forensic psychiatric populations elsewhere (67, 68). The results of the FQL-SV showed that patients at the Veneto REMS were satisfied with the vast majority of QoL aspects (78% of the items had a mean score >50). Moreover, 74% of the patients reported a moderate to high QoL. The aspects patients were least content about were sexuality, nutrition, pleasant environment, and activities. Despite the compulsory nature of admission to the REMS, patients were relatively satisfied, which might also explain the relatively high score on acceptance of residing in a forensic psychiatric unit for a while.

Compared to their clinicians, the patients in our study underreported the number of needs, whether met or unmet. Here it's worth noting that the findings of earlier CANFOR studies are inconclusive. Our results are in line with the findings from Pillay and colleagues (69), who found a structural under-reporting by patients across units with different levels of therapeutic security. Thomas and colleagues (20) and a study by Abou-Sinna and Luebbers (18) also found that patients reported significantly fewer total needs than their professionals. With respect to unmet needs, however, Abou-Sinna and Luebbers (2012) found a non-significant difference, Thomas and colleagues (2008) omitted to report whereas others found that patients reported significantly more unmet needs than their professionals (15, 18, 20, 36). Some of these studies (18, 69), furthermore, reported moderate to strong correlations between patient- and staff-reported needs. In our study, moderate correlations were found for total and unmet needs but not for met needs. Moreover, the level of agreement between patients and clinician in our study was moderate to good on 36% of the identified needs (nine out of 21 Kappa values were > .40) and on 54% of the unmet needs (seven out of 13 Kappa values were > .40). To the best of our knowledge, no CANFOR studies have reported per need the level of agreement between patient and clinician in a forensic psychiatric setting. In general psychiatry, however, comparably low levels of agreement were found (70, 71). Better levels of agreement were found, as might be expected, in areas with a more objective response (e.g. basic education, sexual oppression and childcare).

Higher numbers of patient-rated unmet needs were associated with higher scores on the HCR-20 historical subscale, which is generally considered as the static or actuarial part of the instrument, expressing only fixed, non-modifiable variables. This is an important finding, as patient-reported needs were not related to the HCR-20 clinical and risk subscales, meaning that the correlations are displayed only for past events and problems but not for current or future personal aspects. This supports previous findings regarding the CANFOR (18); namely, that it provides unique information about patients' criminogenic and non-criminogenic treatment needs. Consistent with other studies (18), higher numbers of clinician-rated needs were associated with higher risk, according to the HCR-20 historical and clinical subscales. This might be expected, as both instruments capture the same perspective, namely the patient's current state of recovery according to the clinician. Furthermore, HCR-20^V3^ clinical items investigate the current situation, and are those more contiguous with the CANFOR's treatment needs.

Understanding patients' needs is essential to improving their subjective QoL. Our study showed that a decrease in numbers of needs, and not solely a decrease in unmet needs, reported both by the patient and the clinician, are associated with higher levels of QoL. Likewise, lower numbers of risk factors, specifically those on the HCR-20 historical and clinical subscales, enhances QoL. This result is in line with studies conducted in general psychiatry or outpatient communities (71, 72). Nevertheless, further research is indicated, as this relation seems only longitudinal for patient-rated unmet needs or the social domain of treatment needs (31, 73). Higher numbers of clinician-reported total and unmet needs were associated with lower levels of acceptance of residing in a forensic psychiatric unit for some time. This relation, however, might have been influenced by the treatment phase of a patient. Patients with less (unmet) treatment needs are generally considered closer to discharge from the REMS, which might make it easier to accept their admission than for patients recently admitted. Acceptance of stay, on the other hand, was positively correlated with QoL, meaning that higher levels of acceptance are associated with a more positive QoL appraisal. None of the measures was associated with the duration of time that patients have been admitted to forensic psychiatric services.

Finally, quality of life was assessed with the Italian version of the FQL-SV. Although the original version showed good psychometric properties, the Italian translation had not been validated yet. To that purpose, the Veneto REMS also included the WHOQoL-Bref in the battery of ROM instruments. The internal consistency of the Italian FQL-SV was good; the Cronbach's alpha was 0.86, which was higher than in the original version (28). Construct validity with the WHOQoL-Bref was largely in accordance with the results presented by Schel and colleagues (2016). Positive relations were found between FQL-SV items and WHOQoL-Bref domains that were expected to assess comparable underlying constructs. However, these relations were of moderate magnitude, underlining the assumption that the FQL-SV and WHOQoL-Bref differ in their conceptualization of QoL. Though test-retest reliability would be needed to further validate the FQL-SV, the current study has made it plausible that the FQL-SV is a valid and reliable tool to assess QoL, justifying its use for routinely assessing QoL at the REMS.

### Limitations

The current study has a number of important limitations. First, the number of patients included in this study was small, which might limit its representativeness for the whole forensic psychiatric population in Italy. On the other hand, all REMS consist of small-scale units with maximum of 20 beds and the authors have no reason to believe that there are regional differences in those admitted to the REMS (3). Second, the small population did not allow us to investigate the group differences of needs and QoL, whereas previous research has shown that male and female patients report different needs profiles and various primary diagnoses showed differences in QoL appraisal (33, 34, 37, 74). Third, the data were collected as part of routine care by their treating key-clinicians. Patients might have given desirable responses to convince their clinicians of treatment progress. Fourth, this is the first time the data of the established ROM battery have been analyzed and not all available data were included. Therefore, the current study has provided valuable insights for further development of the Veneto REMS' ROM battery and dataset. Fifth, many patients were admitted at the same time (opening of REMS beginning 2016), this caused that some patients resided already several (3 to 12) months before these first assessments took place. Further development of the ROM should, therefore, also involve establishing fixed assessment moments to be able to link the findings to the different phases of treatment and recovery. Sixth, our study did not include any measure to assess criterion validity. This limits the assumptions that can be made about the (long-term) effect of addressing needs and QoL and how these concepts might contribute to the effectiveness of recovery-oriented treatment.

### Implications for Research

First, the current study could only investigate cross-sectional associations for treatment needs, risk factors, and QoL. Future ROM data will be of longitudinal nature; hence, these data might provide more insight in how meeting needs and QoL improvement might be related to progress in recovery-oriented treatment. Second, our ROM dataset lacked a measure of need for therapeutic security. This could have given information about whether the patients in the REMS are comparable to forensic psychiatric patients elsewhere in Europe (e.g. TBS hospital in the Netherlands or forensic psychiatric hospital in Ireland, the UK or Germany). Third, future research is also needed on more objective indicators in forensic psychiatric settings. Our ROM dataset did not allow us to control for more objective indicators such as leave status, received treatment interventions, treatment phase, level of restrictiveness, social contacts, (aggressive) incidents; some of these aspects might have had an intervening effect on the relationships between needs, QoL, and risk factors. Fourth, more research is needed on the level of agreement between patient- and clinician-rated outcomes. Especially in relation to recovery-oriented treatment, where the therapeutic alliance is key to successful treatment. More insight is needed on the aspects that interfere here in order to improve the level of agreement and facilitate recovery-oriented treatment in forensic psychiatry. On the other hand, recovery-oriented treatment in forensic psychiatry has many challenges (75–79), and empirical evidence on how to deploy the concept is scarce. Nevertheless, the lack of substantial, quantitative research should not imply further postponement of investigating evidence-based recovery-oriented interventions from general psychiatry and how these could be further developed for the forensic psychiatric field.

### Implications for Practice

Far from being a simple humanitarian approach to guarantee a better stay for those admitted, needs and quality of life provide substantial information to support pathways of care and the necessary practice of risk assessment and management. Qualitative studies have shown that recovery in forensic psychiatry, apart from public safety, can have a broad range of treatment outcomes (78). For instance, patients define recovery in terms of a normal, independent, compliant, healthy, meaningful, and progressing life (80). The additional value of addressing needs and QoL, apart from the more obvious ethical reasons such us respect for dignity and rights, may lie in incorporating the patient's perspective. Although the detained status of forensic patients imposes real limits on the capacity for autonomy and choice, incorporating the patient's perspective on decision-making processes, in relation to aspects of treatment, care, and daily life, might have notable benefits. Being involved may give patients a sense of self-efficacy and responsibility, increase their motivation and treatment adherence, improve the therapeutic alliance and give clinicians a better idea of the patients' insight into their risk factors. Nevertheless, here it should be mentioned that our study did not include an outcome measure for successful treatment (e.g. discharge from the REMS). Although needs and QoL assessment might provide additional information for treatment planning, it cannot be concluded that it helps to reduce patients' future risk of recidivism or readmission to forensic psychiatric services. Research on the effectiveness of involving forensic psychiatric patients in their treatment planning is still in its infancies. Some studies found only limited evidence for involving forensic (out-) patients in the decision-making process of risk assessment and management (81, 82), whereas a recent review study (83) showed some favorable support for incorporating patient perspectives, thereby emphasizing the importance of correct instruments to guide the patient-clinician collaboration in risk assessment and treatment planning.

This study has shown that the level of agreement on specific needs between patient and clinician is low. Notwithstanding, this is fundamental in recovery-oriented treatment planning. The level of agreement seems to improve along the different phases of forensic psychiatric treatment, meaning that differences between patient and clinician ratings diminish in concordance with movement to lower levels of security (84). Incorporation of the patient's point of view guarantees a more open and sincere adherence to treatment and care, it emphasizes empowerment and contributes to the recovery of patients (85). Nevertheless, patient-reported outcomes should be interpreted with caution as they might diverge due to cognitive affections, distortions of the perception and low insight, typical of people suffering from chronic mental disorders (86). This is the case for general psychiatry as well as forensic psychiatry, though in the latter more caution is needed as patients might try to influence their legal status through giving desirable responses and presenting a better version of themselves (87).

Finally, some specific aspects deserve attention in forensic psychiatric services. Social needs are frequently unmet in populations with restrictions of personal freedom. Hence, efforts should be made for community interventions enabling patients to get to know people, and improve their social skills and relational abilities. Sexuality in forensic psychiatric services is often neglected or considered complicated by staff and management, and therefore avoided (88). Studies in general psychiatry have shown that half of the patients never spoke or seldom spoke about sexual functioning with their healthcare professionals (89). In forensic psychiatric services, many patients are of an age that is considered critical in an individual's development of adult sexuality and personal relationships. Although policies should be developed in this context, a start could be made by recognizing sexuality as a need and discuss it as part of treatment planning. Another important aspect is satisfaction with daytime or leisure activities, which has been associated with higher QoL (27, 29, 90). However, daytime activities in forensic psychiatry are often characterized by passive leisure (e.g. watching television) and rest (91). Patients feel to only have the choice between participating in occupational activities and refusing to participate, and that refusing to participate could prejudice their discharge possibilities (92). Nonetheless, it is recognized that patients are more likely to enjoy self-chosen occupations; they prefer to spend time engaged in activities that they value, enjoy and feel they do well (91). In line with the recovery paradigm, patients should be consulted regarding their preferences and involved in the organization of activities that fulfill them.

## Data Availability Statement

The datasets generated for this study will not be made publicly available because providing access to these data would be a breach of the GPDR and in conflict with the institutional policies. Requests to access the datasets should be directed to the corresponding author.

## Ethics Statement

The study was approved by the Comitato Etico di Verona (Ethics Committee of Verona).

## Author Contributions

EV and LC conceived of the presented idea. LC amalgamated the collected the data. EV processed the anonymized data, performed the analysis, drafted the manuscript, and designed the figures. LC aided in interpreting the results. Both authors, EV and LC, discussed the results, commented on the manuscript, and contributed to its final version.

## Conflict of Interest

The authors declare that the research was conducted in the absence of any commercial or financial relationships that could be construed as a potential conflict of interest.
